# Effects of Fish Oil Capsules and Vitamin B1 Tablets on Duration and Severity of Dysmenorrhea in Students of High School in Urmia-Iran

**DOI:** 10.5539/gjhs.v6n7p124

**Published:** 2014-09-18

**Authors:** A. Hosseinlou, V. Alinejad, M. Alinejad, N. Aghakhani

**Affiliations:** 1 Urmia University of Medical Sciences, Urmia, Iran; 2 Center of Clinical Research and Reproductive Health, Urmia University of Medical Sciences, Urmia, Iran; 3 Tabriz University of Medical Sciences, Tabriz, Iran; 4 Department of Nursing, School of Nursing & Midwifery, Urmia University of Medical Sciences, Urmia, Iran

**Keywords:** fish oil capsules, vitamin B1, duration, severity, Dysmenorrhea, Urmia, Iran

## Abstract

**Aims:**

The purpose of this study is the comparison of the effect of vitamin B1 and fish oil with together on severity and duration of dysmenorrhea, and if it is effective, we can administrate both of them with less complication to compare with other chemical drugs which has many disadvantages.

**Study Design:**

High school of Urmia city, between March 2008 and June 2008.

**Methodology:**

This study has a double-blind clinical trial design.240 high school female students with dysmenorrhea by a randomized Method were followed up in a double-blind, randomized, placebo-controlled study by dividing into four groups with 60 members. The daily supplement was vit B1 (100 mg/day and fish oil pearl 500 mg/day), taken as a single dose starting at the beginning of the menstrual cycle and continued for 2 consecutive months.

**Results:**

Intensity of pain in three experimental groups (Vit B1, fish oil and both of them) had significant difference comparing placebo group and intensity of pain had reduced. (p<0.001), (p=0.018), (p<0.001) VS in placebo group (p=0.79). Duration of pain had significantly reduced in all three experimental groups compared with placebo group. (p=0.004), (p=0.008), (p<0.001) VS in placebo group (p=0.32). In all of drugs, results (mean) was better at the end of the second month than the first month.

**Conclusion:**

Fish oil and vit B1 effects on treatment of primary dysmenorrhea were similar, but vit B1 has less complications and it was more acceptable. We mixed them and compared its results with vit B1, fish oil and placebo separately. Pain duration is the least in Vitamin B1 tablets compare with the others, but its duration was minimum in complex of Vitamin B1 tablets and fish oil capsules. Due to good effects of vitamin B1 and fish oil on symptoms of dysmenorrhea, using of them is suggested.

## 1. Introduction

Dysmenorrhea is painful contractions which happens before the menses and in 10% of the cases, it cause the person to stop working during daily activities and with financial and social problems (Juli, 2003). Depending on anatomical and pathological cases, dysmenorrhea is categorized into primary and secondary types Primary dysmenorrhea involves menstrual pain without pelvic pathology and is mostly seen in young as the most common gynecologic complaint (Irvani, 2009) that lasts for several years after menarche4. Majority of incidence in adolescents is primary one (O’Connell & Westhoff, 2006).

The risk factors of it are long and heavy menstrual flow, early menarche, and lower consumption of fruits and fish (Balbi et al., 2000).

Analgesics, contraceptives, gonadotropin-releasing hormone agonists are various strategies to cope with dysmenorrhea (Tonini, 2002).

Adolescents should be aware of the relationships among alternative therapies, and relief of menstrual pain to know suitable approaches to improve their quality of life.

Complementary therapies like massage, aromatherapy, and heating or cooling therapy have been practiced for many years (Durain, 2004). Usually, non-steroidal anti-inflammatory drugs (NSAIDs) are used for its treatment (Mehlisch, Ardia, & Pallotta, 2003).

Fish oil can prevent dysmenorrheal symptoms such as abdominal pain and, to some degree, low back pain and headache (Moghadamnia, Mirhosseini, Abadi, Omranirad, & Omidvar, 2010).

In fish oil, Omega-3 fatty acids incorporate into cell wall phospholipids and through competition at the prostaglandin synthetize level may result in the production of less prostaglandin (Hansen & Olsen, 1988; Deutch, 1995).

Vitamin B1 is solvent in water and used in body for muscular and nervous system activities and can affect positively on womb muscular contraction (Zafari, Aghamohammady, & Tofighi, 2011). Many studies show that that vitamin B1 and fish oil reduce symptoms of dysmenorrhea separately and have limited side effects compare with other drugs (Zafari & Aghamohammady, 2011).

Vitamin B1is solvable in water and actives like blood making, carbohydrate metabolism, central nervous system activities and nervous muscle system and so on his almost negative troubles because it has a role in nervous activity and muscles tonus and can be effective on basic dysmenorrheal (Juli & Jolin, 2003).

This study carried on to determine effect of vitamin B1 and fish oil with together on severity and duration of dysmenorrhea, and if it is effective, we can administrate both of them with less complication, to compare with other chemical drugs which have many disadvantages.

## 2. Material and Methods

This study has a double-blind clinical trial design which was carried out between March 2008 and June 2008. A double-blind, randomized, placebo-controlled study carried on 240 high schools female students with dysmenorrhea in Urmia city by dividing into four groups with 60 members. All the participants signed a consent form. The study was carried out based on the principles of the Declaration of Helsinki and approved by Urmia University of Medical Sciences ethics committee. They received drug boxes each month, and for all of them, possible drug complications were described and asked them to mention any occurred complications.

The daily supplement was vit B1 (100 mg/day and fish oil pearl 500 mg/day), taken as a single dose starting at the beginning of the menstrual cycle and continued for 2 consecutive months. Based on previous studies, dosage and the treatment period were selected (Gould, 1996). When menstrual pain occurred; they were not allowed to use any rescue medication while continuing with the experimental protocol. Maximum pain intensity was assessed just before use of supplements. The inclusion criteria included: age 13 to18 years, being single, suffering from dysmenorrhea, having regular menstrual cycles, having no other health problems (according to their medical history), and low dietary fish intake (not more than once per week).The girls were randomly assigned to 1 of 4 schedules. The first group received vitamin a B1 tablet, the second group utilized a fish oil capsule and the third group used a mixture of both fish oil capsules and vitamin B1100 mg tablets and the fourth group received placebo per day from the beginning of menorrheal period duration for two months. We measured severity of pain by Visual Analogue Scale (VAS) (Gould, 1996) that they marked on the line the point that they feel represents their perception of their pain and its duration by Cox Menstrual Scale that can be used to assess the frequency and duration of symptoms of dysmenorrheal (Jafary, 2004), in three times; at the beginning of study, one month and two months after taking the drugs or placebo. The participants were requested to complete a detailed questionnaire assessing their menstrual pain and duration. Collected data were listed and summarized and comparison between and within groups was made by ANOVA test with SPSS software. (Dalfard, Ardakani & Banihashemi, 2011)

## 3. Results

The results showed that intensity of pain in all three experimental groups (vit B1, fish oil and both of them) had significant difference with placebo group and intensity of pain had reduced. (p<0.001), (p=0.018), (p<0.001) VS in placebo group (p=0.79) (Table 1).

**Table 1:** Comparison of severity and duration of pain in the study groups at the before enrollment and after one and two months

Compare the intensity and duration of pain In three interval	Mean(SD)	p	Compare the intensity and duration of pain In three interval	Mean(SD)	p
**Pain intensity in the group receiving vitamin B**		<.001	**Pain intensity in the group receiving fish oil**		.018
**Before enrollment**	7.49(2.15)		Before enrollment	7.59(2.15)	
**One month after treatment**	4.11(1.73)		One month after treatment	5.22(1.96)	
**Two months after treatment**	2.38(1.50)		Two months after treatment	3.14(1.42)
**Duration of pain in the group receiving vitamin B**		.004	**Duration of pain in the group receiving fish oil**		.008
**Before enrollment**	37.98(46.1)		Before enrollment	11.0(86.7)	
**One month after treatment**	20.225(75.24)		One month after treatment	7.21(27.23)	
**Two months after treatment**	22.26(81.17)		Two months after treatment	98.16(43.19)
**Pain intensity in patients who received placebo**			**Pain intensity in the group receiving Combination therapy**		<.001
**Before enrollment**	7.49(2.06)	.791	Before enrollment	7.39(1.02)	
**One month after treatment**	73.1(81.6)		One month after treatment	4.01(1.20)	
**Two months after treatment**	50.2(28.7)		Two months after treatment	2.29(1.79)
**Duration of pain In the group receiving placebo**		.322	**Duration of pain in the group receiving Combination therapy**		<.001
**Before enrollment**	38.45(37.7)		Before enrollment	38.02(27.11)	
**One month after treatment**	25.32(46.36)		One month after treatment	11.36(33.20)	
**Two months after treatment**	24.38(76.37)		Two months after treatment	72.56(91.41)	

Duration of pain had significantly reduced in all three experimental groups compared with placebo group. (p=0.004), (p=0.008), (p<0.001) VS in placebo group (p=0.32).

Table 1 shows the intensity and duration of pain in the study groups before enrollment compare with two months after treatment.

Table 2 shows the intensity and duration of pain in the study groups compare with the placebo group in three period of time.

**Table 2:** Comparison of severity and duration of pain in the study groups and the placebo group at three period of time

Comparison of pain duration between study groups and placebo in three period of time		Mean (SD)	p	Comparison of pain intensity between study groups and placebo in three period of time		Mean (SD)	p
Fish Oil VS Placebo group	Before enrollment Placebo group	37.16(30.11) 38.45(37.7)	.834	Fish Oil VS Placebo group	Before enrollment Placebo group	7.59(2.15) 7.49(2.06)	**.791**
	One month after treatment Placebo group	23.27(21.7) 36.46(32.25)	.004		One month after treatment Placebo group	5.22(1.96) 6.81(1.73)	**.001**
	Two months after treatment Placebo group	19.43(16.98) 37.76(38.42)	.007		Two months after treatment Placebo group	3.14(1.42) 7.28(2.50)	**.001**
Combination therapy VS Placebo group	Before enrollment Placebo group	38.02(27.11) 38.45(37.7)	.631	Combination therapy VS Placebo group	Before enrollment Placebo group	7.39(1.02) 7.49(2.06)	**.711**
	One month after treatment Placebo group	20.33(36.11) 36.46(32.25)	.013		One month after treatment Placebo group	4.01(1.20) 6.81(1.73)	**<0.001**
	Two months after treatment Placebo group	14.91(56.72) 37.76(38.42)	.008		Two months after treatment Placebo group	2.29(1.79) 7.28(2.50)	**.001**
Vitamins B 1 VS Placebo group	Before enrollment Placebo group	37.98(46.1) 38.45(37.7)	.842	Vitamins B 1 VS Placebo group	Before enrollment Placebo group	7.49(2.06) 7.49(2.06)	**.634**
	One month after treatment Placebo group	24.75(20.25) 36.46(32.25)	.037		One month after treatment Placebo group	4.11(1.73) 6.81(1.73)	**.001**
	Two months after treatment Placebo group	17.81(37.7) 37.76(38.42)	.037		Two months after treatment Placebo group	2.38(1.50) 7.28(2.50)	**<0.001**

The intensity of pain during two months of the intervention showed a statistically significant decrease between the three groups compared with the placebo group (p<0.001) (Table 2).

The duration of pain in the experimental groups compared with the placebo group during two months of the intervention showed a statistically significant decrease in all groups (P<0.05) (Table 2).

In all of drugs, results (mean) was better at the end of the second month than the first month except for the fish oil capsules usage and its severity was the least in both supplement usage. There is a significant difference between drug results with placebo results. The significance level was set at (p< 0.05).

## 4. Discussion

We compared the medical effect of vitamin B1 and fish oil and combination therapy in relief the pain intensity and duration of dysmenorrheal in girls.

The result showed the pain intensity in experimental groups compare with the placebo group had significant decrease. In Wilson and Murphy (2001) study, consumption of fish oil decreases the primary and secondary dysmenorrheal (Wilson & Murphy, 2001).

In a clinical trial study, the effect of vitamin B1 and fish oil on the treatment of primary dysmenorrhea were compared and the results showed that fish oil and vit B1 effects on treatment of primary dysmenorrhea were similar, but vit B1 has less complications and it was more acceptable as like as another research (Zafari, 2011). A research results showed the effect of vitamin B1 on dysmenorrhea. It was evaluated in which the progress percentage was 20% in the first month, 55.6% in the second month and 84.4% in the third month (Nasiri, 2008). Other study showed that the efficacy of fish oil is good in severe pain in dysmenorrheal (Juli & Jolin, 2003). But, in our study we mixed them and compared its results with vit B1, fish oil and placebo separately.

In our study, duration of pain in experimental groups compare with the placebo group had significant decrease.

In the study of Zamani et al. (2001), fish oil decreased the duration of dysmenorrheal pain after two month of administration (Zamani & Soltanbeighi, 2001). Also in French study (2005), fish oil decreased the duration of dysmenorrheal pain and 60 percent of the participants were satisfied of fish oil consumption (French, 2005).

Duration of pain was the least in Vitamin B1 tablets compare with the others, but its duration was minimum in complex of Vitamin B1 tablets and fish oil capsules. To compare the present study result with the other studies, it can be said that the medical effect of vit B1 and fish oil is good, but the medical troubles of non-steroid drugs are very high and sometimes cause to cut the medicine usage by the patient (Nasiri, 2008).

## 5. Conclusion

Dysmenorrhea is a debilitating condition for women in their reproductive age. Supplements as a multidisciplinary approach should be used to limit the impact of this condition on activities of daily living and quality of life. Due to good effects of vitamin B1 and fish oil on symptoms of dysmenorrhea, and stand ability which can be substituted instead of high complication medicines of no steroid anti-inflammatory to cure this disease for the people suffered from it. Further research, however, using higher amount doses of fish oil and vitamin B1 in all cycle length, more time and longer treatment periods is recommended.
